# Perforated gastric ulcer post mini gastric bypass treated by laparoscopy: A case report

**DOI:** 10.1016/j.amsu.2019.11.006

**Published:** 2019-11-17

**Authors:** Diego Paim Carvalho Garcia, Cyntia Ferreira dos Reis, Luiza Ohasi de Figueiredo, Guilherme Vaz de Melo Mota, Leonardo Quinete Guimarães, Fernando Augusto de Vasconcellos Santos, Luiz Ronaldo Alberti, Thiago de Almeida Furtado

**Affiliations:** a Department of Surgery, Hospital Felício Rocho, Av. Do Contorno, 9530 – Barro Preto, Belo Horizonte, MG, 30110-934, Brazil; b Hospital Felício Rocho, Av. Do Contorno, 9530 – Barro Preto, Belo Horizonte, MG, 30110-934, Brazil; c Hospital Alberto Cavalcanti, R. Camilo de Brito, 636 – Padre Eustáquio, Belo Horizonte, MG, 30730-540, Brazil; d Instituto de Ensino e Pesquisa da Santa Casa de Belo Horizonte, Programa de Pós-Graduação Em Clínica Médica/Biomedicina, Avenida Francisco Sales, 1.111 9 Andar, Ala D - Santa Efigênia, Belo Horizonte, MG, 30140040, Brazil

**Keywords:** Case report, Mini-gastric bypass, Bariatric surgery, Peptic ulcer, Perforated ulcer, Laparoscopy

## Abstract

**Introduction:**

Among the many techniques available for bariatric surgery, the Mini Gastric Bypass is a safe, technically simple and effective option. However, it may present with postoperative complications, being the perforated gastric ulcer one of the most relevant ones.

**Presentation of case:**

A female patient of 41 years of age, with past medical history of a laparoscopic MGB performed 2 year before, presented with 12 hours of sharp and abruptly initiated abdominal pain, with diffuse presentation with suspected perforated acute abdomen after initial medical assessment and examination. Imaging propaedeutic was performed and confirmed a small pneumoperitoneum the patient was submitted to a laparoscopy with closure of the leak and omental patch (Graham's patch) after a thorough abdominal irrigation with saline solution. The patient was discharged from the hospital on the fourth day after surgery.

**Discussion:**

One of the most common complications after and MGB surgery is the occurrence of gastric ulcers and main manifestation of the anastomotic marginal ulcers (MU) is the perforation. The treatment of the perforated peptic ulcer can be performed via laparoscopic or laparotomic approach. The main objective, regardless of the method used to access the abdominal cavity, is to identify and close the perforation.

**Conclusion:**

The perforated gastric ulcer is a complication of the mini bariatric bypasses, and the laparoscopic treatment of the perforation associated with thorough irrigation for of the abdominal cavity and omentoplasty present good results for management of this complication.

## Highlights

• Perforated acute abdomen in 47 year old female post Mini Gastric Bypass (MGB).
• Gastric ulcer is a possible complication of MGB.
• Main manifestation of the anastomotic marginal ulcer is the perforation.
• Laparoscopic treatment with irrigation abdominal and omentoplasty has good outcomes.

## 1 Introduction

Bariatric surgery is an effective option for the treatment of morbid obesity, mostly when no satisfactory result was obtained by clinical treatment. The mini gastric bypass (MGB) is considered a safe, simple and as effective surgical technique as the traditional gastric bypass method with Roux-en-Y reconstruction (RYGB) for treating morbid obesity and Diabetes Mellitus II (DMII) [1]^.^ Some publications even suggest better advantages of the MGB method regarding post operative results, mentioning an important loss of weight in the first two years and high rates of DMII remission, within a smaller procedure time [8]. It is, also, a technique that can be more easily reversed than the other available, such as the sleeve gastrectomy, and less technically intricate than the RYGB. All the advantages of the MGB technique are corroborated by the definitions of the International Diabetes Federation for and appropriate metabolic surgical procedure [1]. This technique differs from the others, and may be beneficiary, due to the performance of a single anastomosis during the surgical procedure. However, the technique has been under scrutiny for the incidence of late onset gastric ulcers, that progress to perforation, and ways to avoid such complication, as well as the most adequate manner to conduct patients that develop such complication and its outcomes.

This paper presents the case of a patient post MGB, admitted in a private tertiary healthcare facility, presenting with a perforated gastric ulcer and a discussion of the available literature on the subject.

## 2 Case report

**Image 1:**
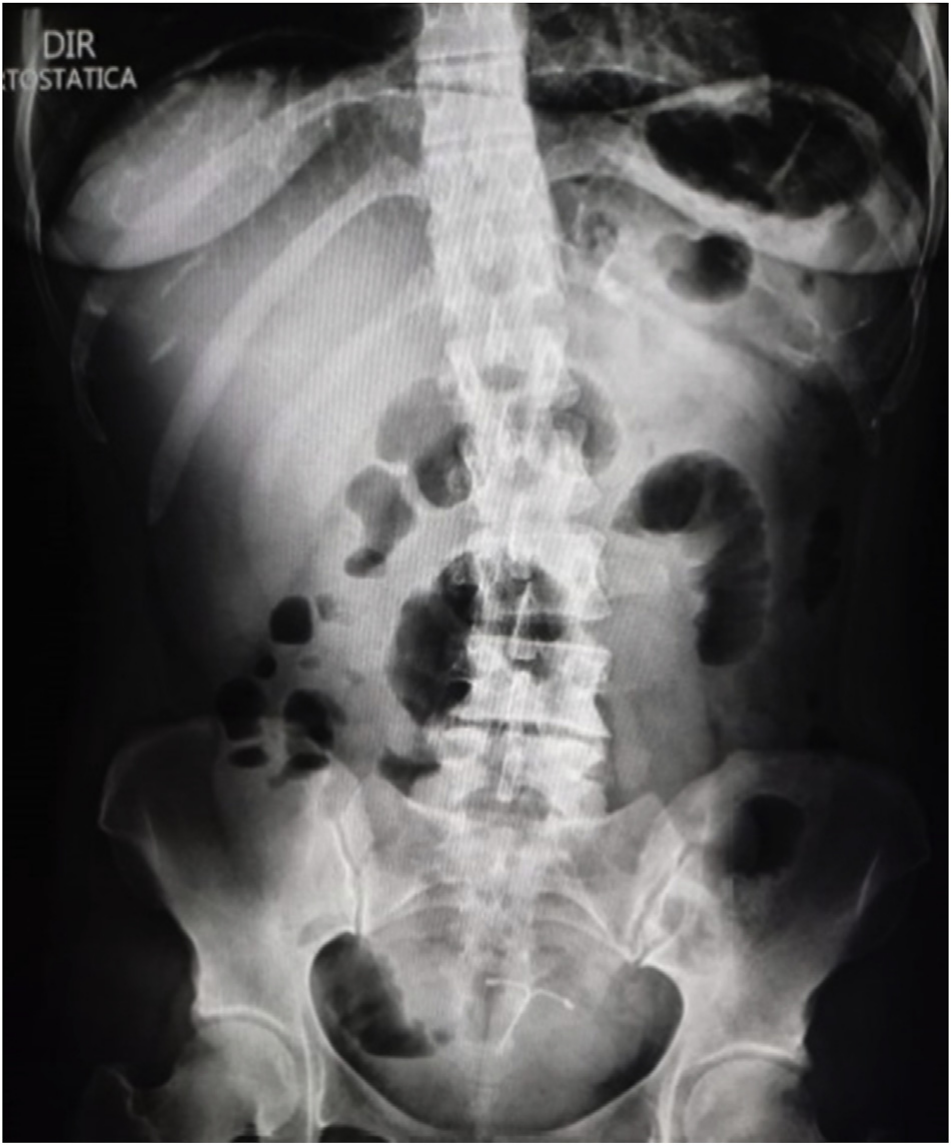
Abdominal Xray showing signs of pneumoperitoneum.

**Image 2:**
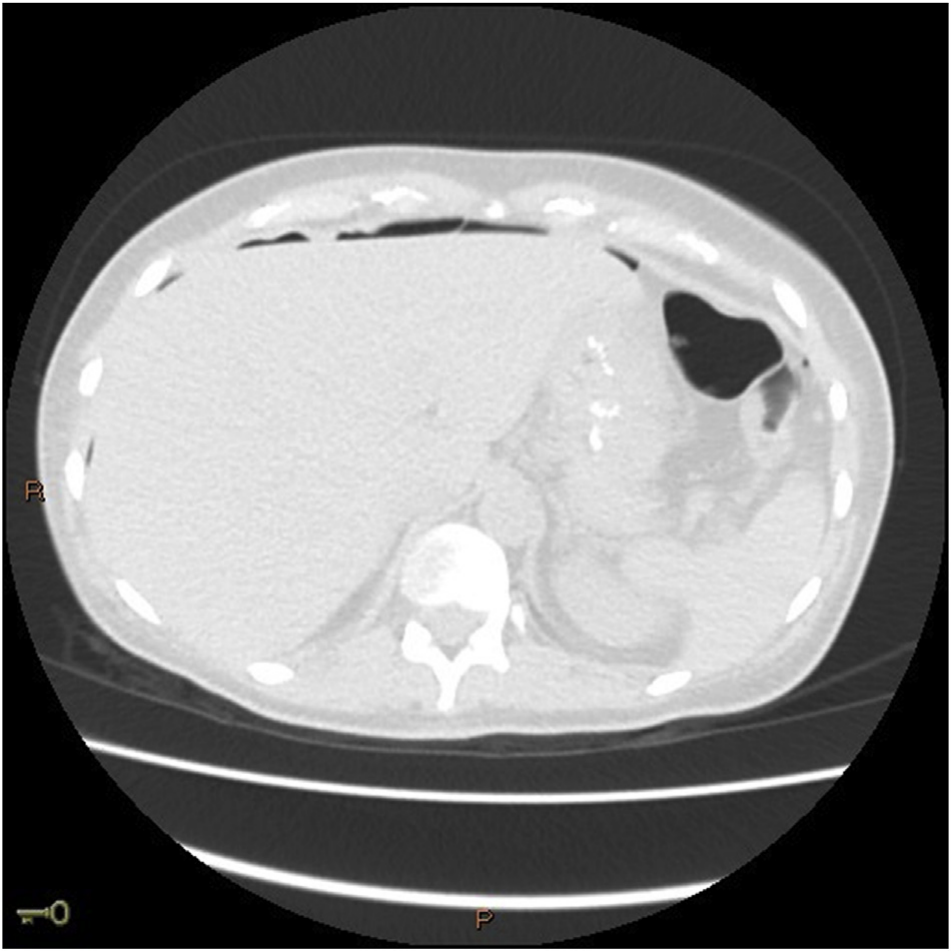
CTscan showing signs of pneumoperitoneum.

**Image 3:**
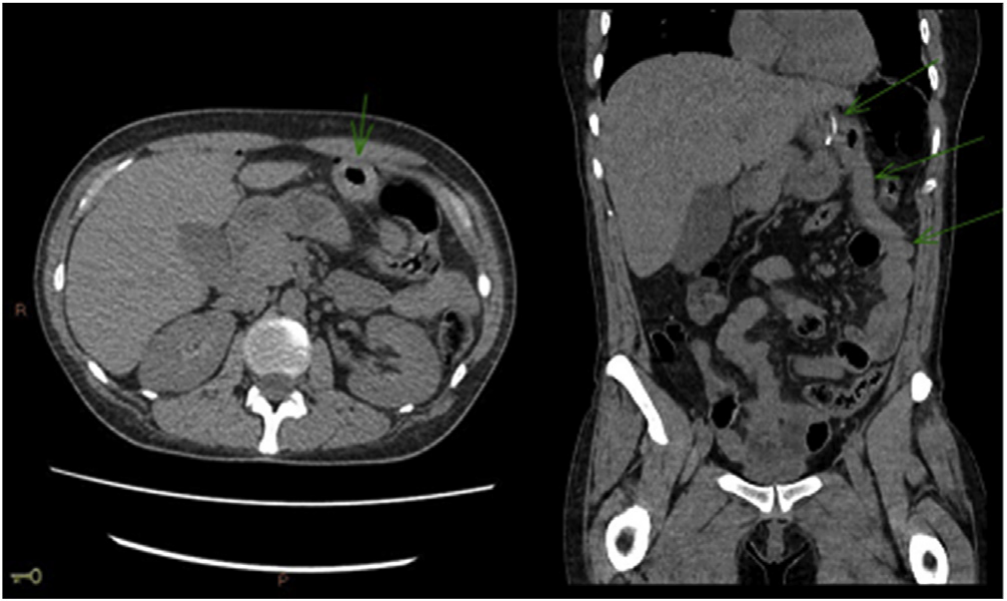
CTscan showing thickening of the ascending jejunal loop.

A female patient of 41 years of age sought medical care after presenting with 12 of abdominal pain. The pain started abruptly and sharp, with diffuse presentation but slightly stronger in the left hypocondrium, periumbilical and epigastric regions, associated with one episode of vomiting. The patient stated that the pain progressed intensity and she developed hiporexia and diarrhea after a few hours. The patient has past medical history of a laparoscopic MGB performed 2 year before, as well as eventual consumption of alcohol and nicotine. She denied recent intake of *anti*-inflamatory medications. She was hemodinamically stable, afebrile, but with a diffuse abdominal rigidity with diffuse peritoneal irritation signs after initial medical assessment and examination. She was submitted to and abdominal radiography (Xray), with signs of pneumoperitoneum (Image 1). She was then conducted to a computed tomography (CT) scan that showed a small pneumoperitoneum localized close to the anastomosis of the previous MGB (Image 2), as well as a thickening of the ascendant jejunal loop, leading the medical team into the conclusion of a perforated acute abdomen due to perforated gastric ulcer (Image 3).

She was then conducted to a computed tomography (CT) scan that showed a small pneumoperitoneum localized close to the anastomosis of the previous MGB (Image 2), as well as a thickening of the ascendant jejunal loop, leading the medical team into the conclusion of a perforated acute abdomen due to perforated gastric ulcer (Image 3).

**Image 4:**
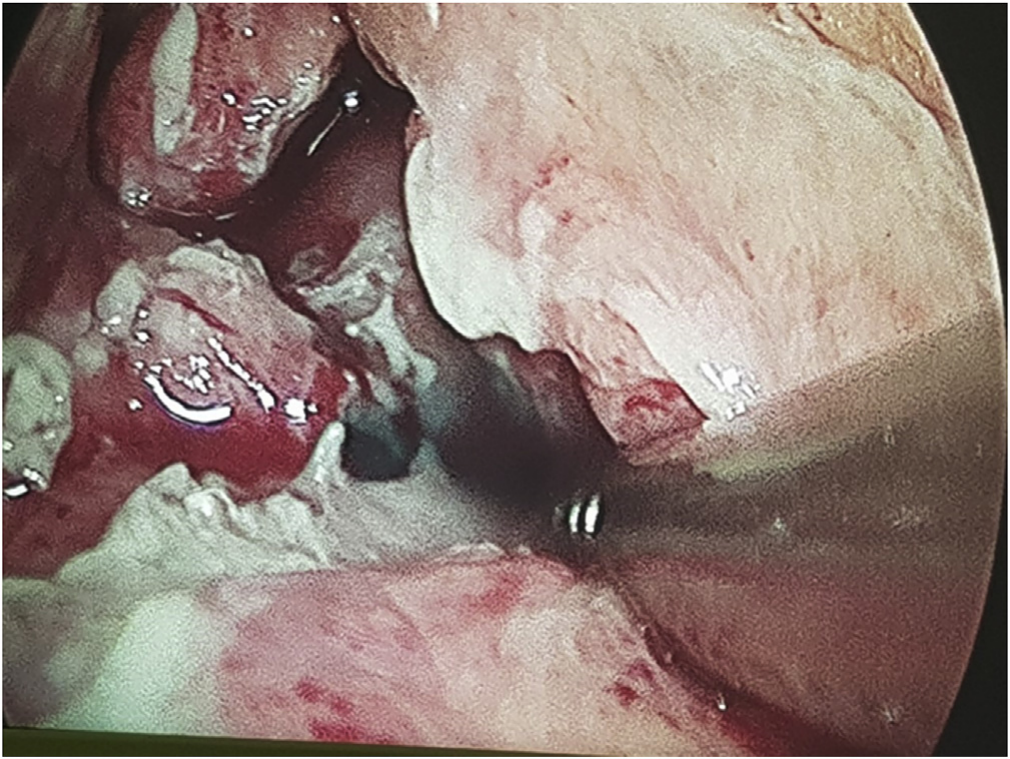
Methylene blue dye leak at the medial portion of the gastro-enteral anastomosis. (For interpretation of the references to colour in this figure legend, the reader is referred to the Web version of this article.)

The patient was taken immediately into surgery, where it was opted by a laparoscopic approach of the above mentioned complication. During the initial assessment of the abdominal cavity an area of fibrin concentration around the gastro-enteral anastomosis, without a clear visualization of a perforated area. A methylene blue dye test was then performed, with the administration of the solution per orogastric tube, with a resulting leak of the dye through a perforated gastric ulcer located at the medial portion of the gastro-enteral anastomosis (Image 4). The leaked point was closed with 3–0 PDS and patched with a portion of the omentum (Graham's patch) after a thorough abdominal irrigation with saline solution. The patient remained hemodinamically stable during the procedure and post-operative period, being discharged from the hospital on the fourth day after surgery.

## 3 Discussion

**Image 5:**
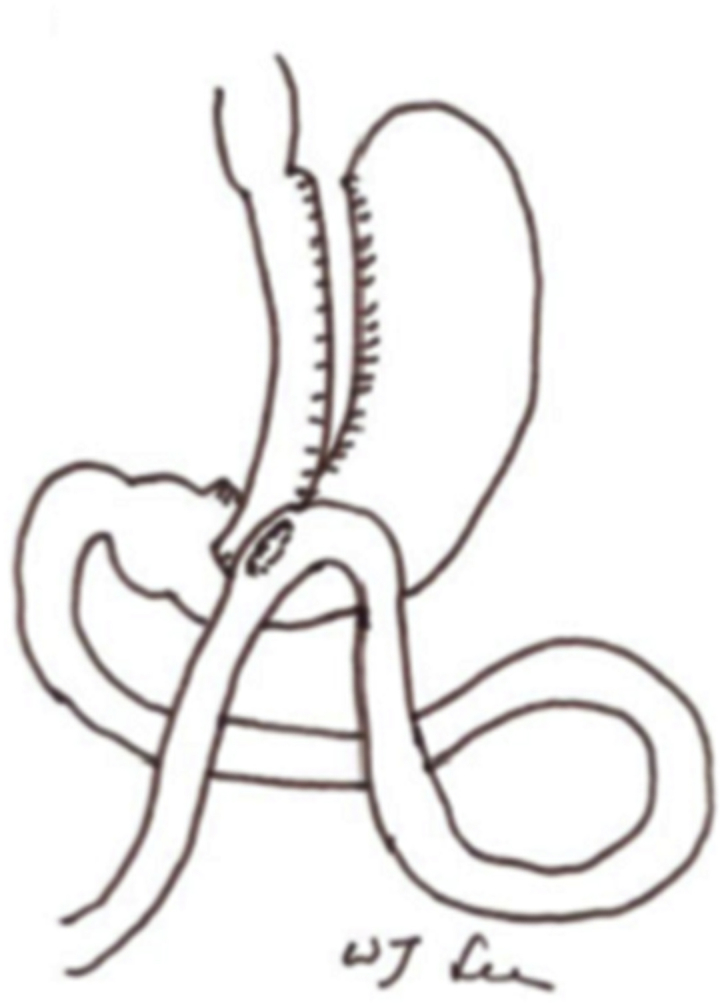
Illustration of a MGB from laparoscopic Roux-en-Y vs. Mini-gastric bypass for the treatment of morbid obesity: a 10-year experience.

The MGB was first described by Rutledge in 1997 and consists of a long intestinal loop being brought from below the crow's foot extending and being attached to the stomach up to the left of His' angle (Image 5). It differs from the RYGB and Sleeve techniques due to its wide gastro-jejunal anastomosis to an anti-colic loop of jejunum, brought upwards 150–200 cm after the ligament of Treitz. The MGB procedure is considered a “Non-Obstructive” restrictive procedure associated with a minimal malabsorption, regardless of the description of significant fatty food intolerance [6].

One of the most common complications after and MGB surgery is the occurrence of peptic ulcers. The incidence of marginal ulcers after a bariatric surgery ranges from 0.6 to 16%, and affects mostly the gastro-jejunal anastomosis. Among the risk factors that take part into the development of the gastric ulcers in such patients the following must be taken into consideration: the presence of DMII, smoking (due to its mechanism of direct microvascular and mucous barrier lesion), a large gastric pouch (presence of residual of parietal cells), gastric fistulas, the use of non-absorbable sutures, use of non steroid *anti*-inflamatory or systemic steroid medications (both reduce prostaglandines within the gastrointestinal tract) and *H. pylori* infection [4]. In the reported case, besides intermittent smoking, the patient denied recent history of *anti*-inflamatory use, and the presence of *H. pylori* was unknown.

The incidence of marginal ulcers was investigated in a study developed by Mark et al. and stated that after a RYGB procedure such incidence ranged 0,85% around 24 months after procedure and 72,2% of the patients presented with the complication were women [4]. However, according to K. K. Mahawa et al. study, showed that the patients submitted to an MGB surgery had no increased rates of marginal ulcers when directly compared to the patients submitted to RYGB surgery, regardless of the assumption that the anastomosis of the previous is constantly exposed to pancreatic and biliary fluids. Other technical differences between both procedures, such as the reduced anastomotic tension due to larges gastric pouch, as well as gastric juices neutralization by pancreatic juice and bile, do not promote a statistical improvement on the incidence of the discussed complication [3].

The main manifestation of the anastomotic marginal ulcers (MU) is the perforation [4]. According to a publication written by Søreide and collaborators, in 2006 over 150000 patients were hospitalized in the United States due to complicated peptic ulcers, with around 4% (14500) of those patients suffering from perforated ulcers. The perforation of a gastric ulcer is considered a common complication with a mortality rate reaching 30% of its subjects [5]. In most cases the patient presents with acute abdominal pain associated with signs of localized or diffused peritoneal irritation and abdominal rigidity, with a high risk of fast lethal progression due the development of sepsis. The immediate surgical approach and early treatment of the sepsis is substantial for a good outcome.

The treatment of the perforated peptic ulcer can be performed via laparoscopic or laparotomic approach. The main objective, regardless of the method used to access the abdominal cavity, is to identify and close the perforation. For elective surgical approaches it is observed that the laparotomy triggers a greater inflammatory response when compared to laparoscopy. The first laparoscopic repair was performed in 1989 and published by Mouret et al. with results showing that this approach could reduce postoperative wound problems and adhesions. The laparoscopic and laparotomic approaches present with similar rates of postoperative complications, necessity of reoperation and mortality. No remarkable statistical difference is identified when compared the length of hospitalization, duration of procedure and introduction of oral diet in patients submitted to either open or laparoscopic repair of perforated peptic ulcers. However, it is proven that laparoscopy reduces the rates of surgical site infection, shorter nasogastric tube duration and reduces postoperative pain (and therefore necessity of opiods) [2].

The treatment of the ulcers, rather via laparoscopy or laparotomy, with irrigation of the abdominal cavity and omentoplasty is considered both safe and effective [4]. A previous research made with 86 surgeons who performed 27672 MGB cases (also known as One Anastomosis Gastric Bypass – OAGB) showed and incidence of 2,24% of MU (622 cases os anastomotic ulcer with or without perforation). Most of the patients underwent gastric endoscopy for diagnostic confirmation, and almost all of the cases of perforation of the ulcers (49 out of 55) were treated laparoscopically with optional omentoplasty and cavity drainage. A small percentage of the cases of non healing ulcers were treated by converting MGB technique to RYGB, they were 46,5% of the cases of patients with MU [3].

There are no recent studies that show benefits regarding a prolonged prophylaxis with proton pump inhibitors in bariatric patients [4]. Some studies suggest a slight reduction of the MU in patients undergoing therapy with pantoprazole 40mg daily for 6 months, with an improvement of MU incidence of 1.2% in the prophylaxis group compared to 7.3% in historical control without prophylaxis [3].

## 4 Conclusion

The perforated gastric ulcer is a complication of the mini bariatric bypasses, and the laparoscopic treatment of the perforation associated with thorough irrigation for of the abdominal cavity and omentoplasty present good results for management of this complication.

## Provenance and peer review

Not commissioned, externally peer reviewed.

## Consent

Written consent was obtained from the patient to publish the case report and all images associated.

## Ethical approval

This paper required no ethical approval.

## Sources of funding

No sources of funding.

## Author contribution

Diego Paim de Carvalho Garcia: Conceptualization, Supervision, Writing – Review & Editing.

Cyntia Ferreira dos Reis: Data Curation, Resources, Writing – Original Draft, Writing – Review & Editing.

Luiza Ohasi de Figueiredo: Writing – Original Draft, Writing – Review & Editing, Visualization.

Guilherme Vaz de Melo Mota: Resources, Writing – Original Draft.

Leonardo Quinete Guimarães: Resources, Writing – Original Draft.

Fernando Augusto de Vasconcellos Santos: Supervision, Writing – Review & Editing.

Luiz Ronaldo Alberti: Conceptualization, Supervision, Writing – Review & Editing.

Thiago de Almeida Furtado: Conceptualization, Supervision, Methodology, Writing – Review & Editing.

## Trial registry number

1. Name of the registry:

2. Unique Identifying number or registration ID:

3. Hyperlink to the registration (must be publicly accessible):

## Guarantor

Thiago de Almeida Furtado.

Diego Paim de Carvalho Garcia.

## Declaration of competing interest

No conflicts of interest.
